# Extranodal Manifestation of Chronic Lymphocytic Leukemia: A Case of Bilateral Breast Involvement

**DOI:** 10.1002/ccr3.70559

**Published:** 2025-05-30

**Authors:** Ensiyeh Bahadoran, Freidoon Solhjoo, Fatemeh SamieeRad

**Affiliations:** ^1^ Cellular and Molecular Research Center Research Institute for Prevention of Non‐Communicable Disease, Qazvin University of Medical Sciences Qazvin Iran; ^2^ Department of Pathobiology Faculty of Medical School, Qazvin University of Medical Sciences Qazvin Iran

**Keywords:** biopsy, breast neoplasms, chronic lymphocytic leukemia, extranodal involvement, leukemic infiltration

## Abstract

Mammography revealed a large, lobulated, hyperdense mass in the right breast occupying nearly the entire breast and axillary region. A smaller but similar lobulated mass was present in the upper outer quadrant of the left breast. BIRADS 4 classification was assigned to both breasts, indicating suspicious findings requiring further evaluation. Based on histopathologic evaluation, it was found that the breast was involved by chronic lymphocytic leukemia.

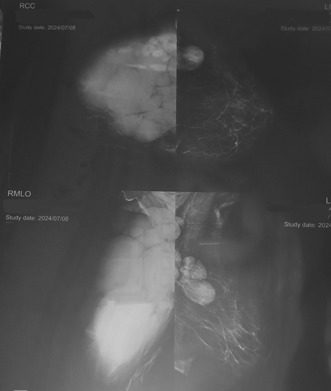

## 1 Introduction

Chronic lymphocytic leukemia (CLL) is a cancer of CD5^+^ B cells. It is defined as the build‐up of small, mature‐looking lymphocytes in the blood, bone marrow, and lymphoid organs [1]. Most CLL diagnoses occur in patients aged 65–74 years. The male diagnoses exceed those of the females by nearly double. It has an incidence rate of five new cases per 100,000 people [2]. The diagnosis is made by ≥ 5.0 × 10^9^/L monoclonal B lymphocytes and a blood film that shows small, mature‐appearing leukemia cells, smudge cells, and lymphocytosis [3]. The clinical presentation of CLL varies greatly; 25% of individuals show up with asymptomatic isolated lymphocytosis. Others exhibit hepatosplenomegaly along with waxing and waning lymphadenopathy, while 5%–10% experience symptoms such as fever, exhaustion, and weight loss. Less frequently, signs of autoimmune or acquired immunodeficiency are observed. Less than 10% of the individuals initially have nodal involvement [4]. Extramedullary and extranodal infiltration is possible with a frequency of 0.3 per 100,000 individuals. The skin and central nervous system are the most often involved extranodal locations in leukemia; however, other organs, such as the liver, lungs, kidney, gastrointestinal tract, bone, prostate, and heart, are sporadically involved [5].

There is an increased risk of developing other cancers in CLL patients. A retrospective study revealed that the risk of skin cancer was eight times higher, the risk of all malignancies was three times higher, and the risk of all cancers except skin cancer was two times higher in patients with CLL than in a control group [6]. Of 2028 CLL patients, 16% had a prior diagnosis of another malignancy, and 11.2% had more tumors discovered during the follow‐up period. The three most prevalent malignancies were breast cancer (9%), prostate cancer (13%), and skin cancer (30%) [6]. The most common tumors that spread to the breast tissue are melanoma, lung cancer, lymphoproliferative disorders, and gynecological cancers (ovary and uterus) [7].

The most prevalent type of primary cancer among women is breast cancer, and it is rarely secondary to leukemia, especially when it is caused by CLL [8]. When CLL infiltrates the breast tissue, it can mimic primary breast malignancies, both clinically and radiologically, leading to diagnostic dilemmas. The final diagnosis can be confirmed via pathological examination [9]. In this report, we present a case of a patient with known CLL who developed breast involvement. This case highlights the need for clinicians to consider leukemic infiltration in patients with CLL who present with new breast masses. The report also underscores the importance of a multidisciplinary approach in such cases for radiologists, gynecologists, and pathologists by combining clinical, radiological, and pathological findings to arrive at a correct diagnosis and determine the most appropriate therapeutic strategy.

## 2 Case History/Examination

A 72‐year‐old female with a history of CLL and a negative history of breast cancer presented with stiffness and a mass sensation in the right breast. Physical examination revealed a large hard mass in the right breast. The right axillary lymph nodes were not enlarged, and no associated skin changes were observed.

## 3 Investigations, Treatment and Differential Diagnosis

**FIGURE 1:**
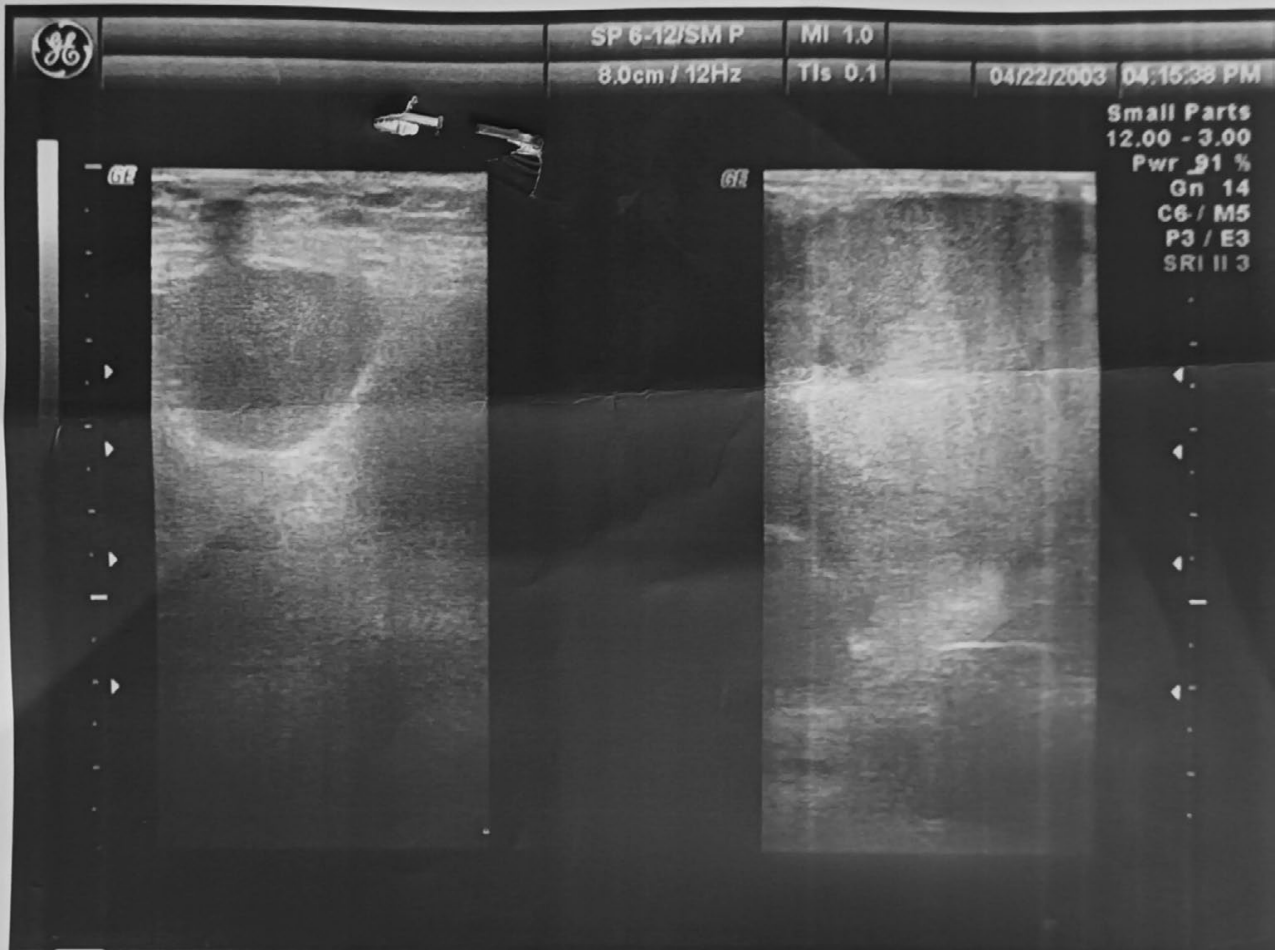
Radiologic evaluation of the breast involvement by CLL showed most of the right breast is surrounded by a relatively uniform hypoechoic solid mass.

On sonography, most of the right breast was surrounded by a relatively uniform hypoechoic solid mass, which had a hypervascular appearance in the color Doppler image. Multiple lymphadenopathies were seen in the right axilla. The above evidence suggests the presence of a neoplastic mass in the right breast. The left fibroglandular tissue was normal. No left‐sided adenopathy was observed (Figure 1).

**FIGURE 2:**
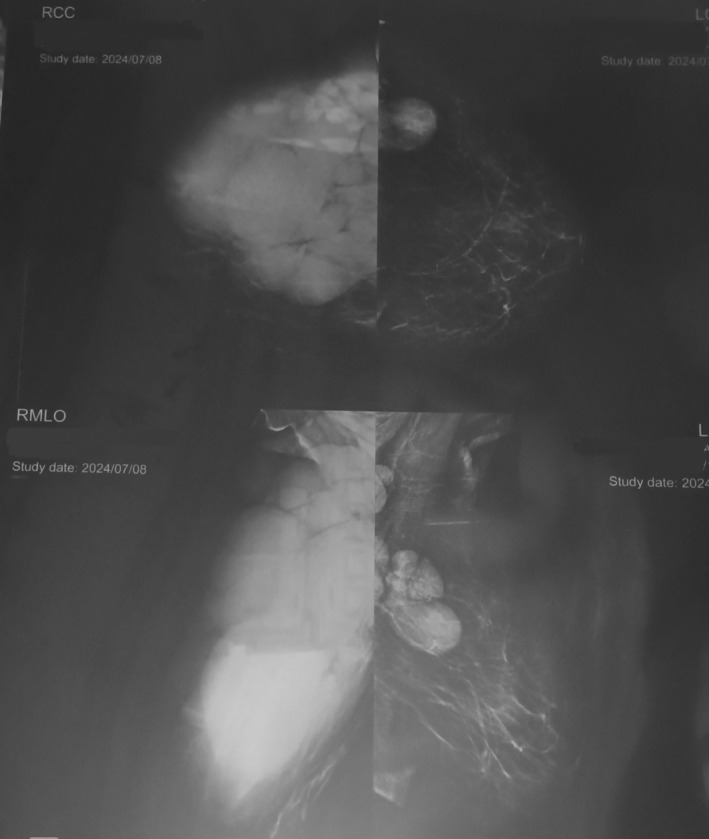
MRI evaluation of breast involvement by CLL showed a giant lobulated hyperdense mass in the right breast, which occupies almost all of the breast and axillary space. Also, a similar, smaller lobulated mass in the upper outer quadrant of the left breast was seen.

Bilateral full digital mammography showed a giant lobulated hyperdense mass in the right breast, which almost occupied the entire breast and axillary space, and only a thin layer of intact breast tissue in the lower inner quadrant of the breast was present. Additionally, a similar, smaller, lobulated mass was observed in the upper outer quadrant of the left breast. A few hyperdense masses were present in the left axillary region, which may be representative of infiltrated lymph nodes. BIRAD 4 was detected in the left and right breasts (Figure 2).

**FIGURE 3:**
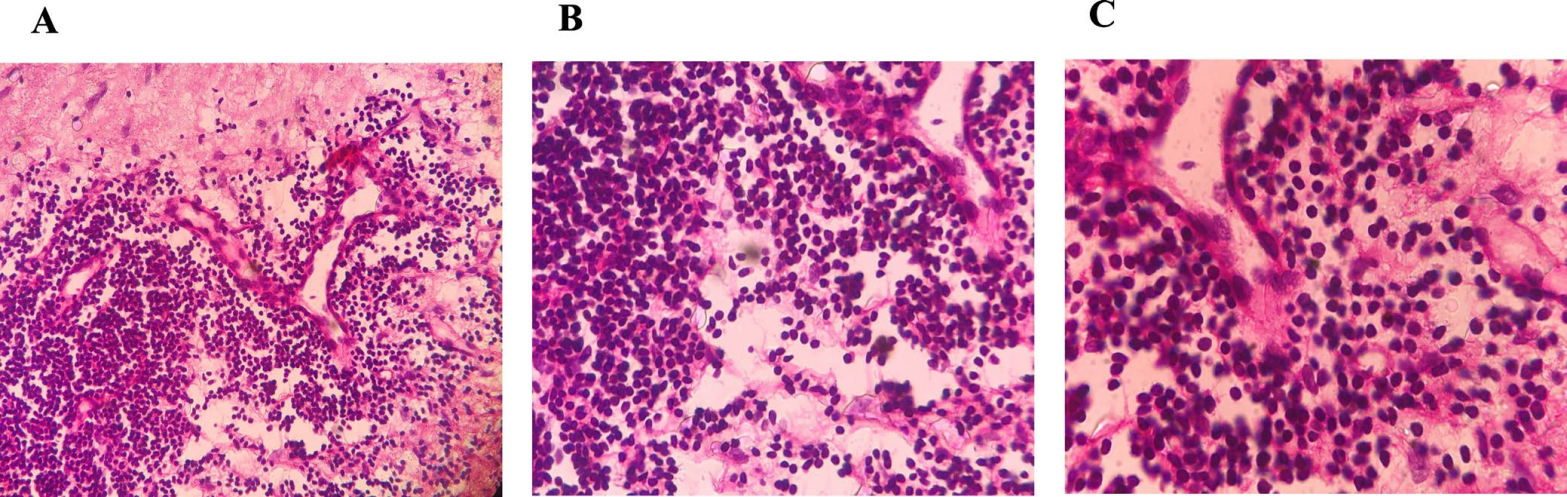
Breast tissue infiltration by lymphocytes with a background of fibrofatty connective tissue, (A) (200×), (B) (400×), (C) (1000×), Hematoxylin & Eosin.

The above findings were suggestive of malignancy, and a core needle biopsy was recommended. Core needle biopsy showed the replacement of solid sheets of tumor cells with a background of fibrofatty connective tissue in the breast tissue. Moreover, tumor cells included small dark round lymphocytes in the lesion, confirming the involvement of the breast with CLL (Figure 3A–C).

**FIGURE 4:**
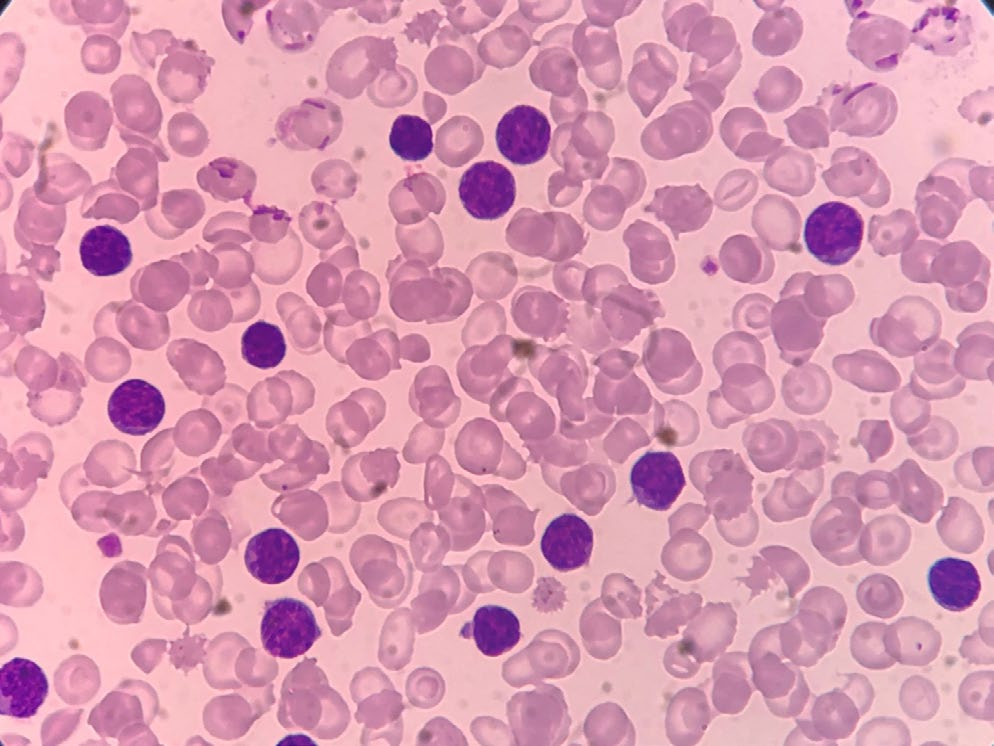
Peripheral blood smear. Peripheral blood smear showing small mature lymphocytes, (1000×).

**FIGURE 5:**
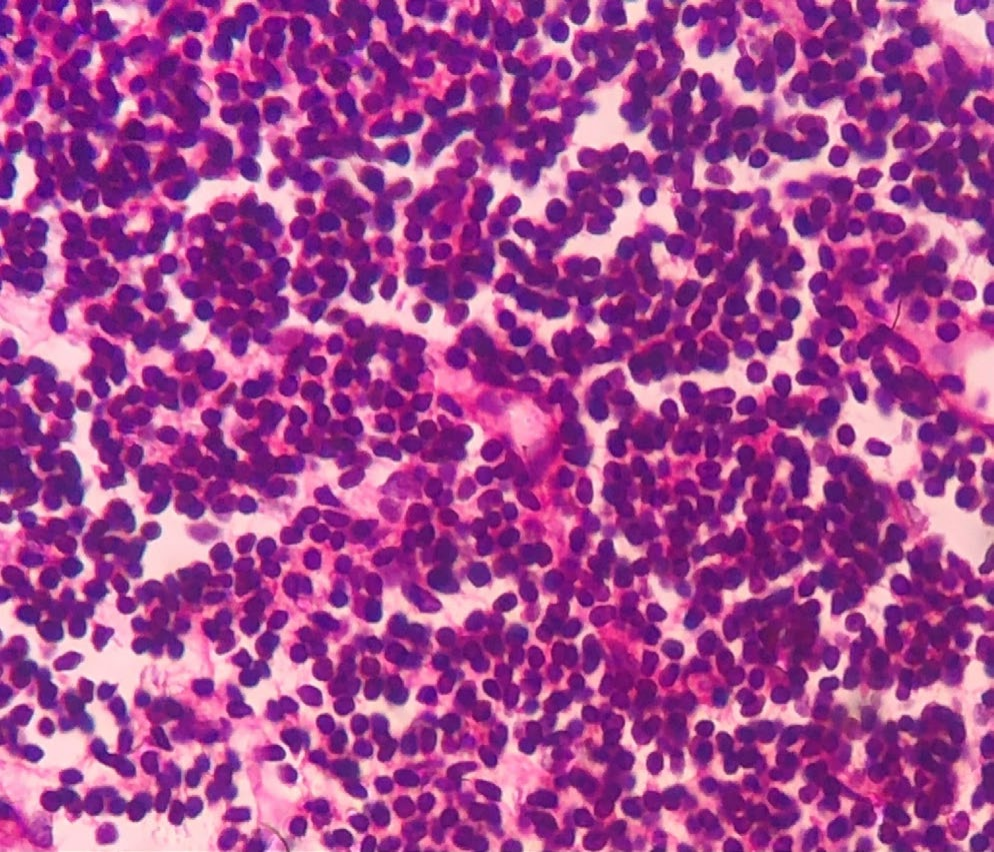
Bone marrow aspirate. Bone marrow aspirate showing lymphocytic infiltration consistent with CLL (200×).

A peripheral blood smear (PBS) was evaluated and showed small lymphocytes with round nuclei and scant cytoplasm, further supporting the diagnosis of CLL (Figure 4). Moreover, the bone marrow aspirate revealed diffuse infiltration by small mature lymphocytes (Figure 5).

**FIGURE 6:**
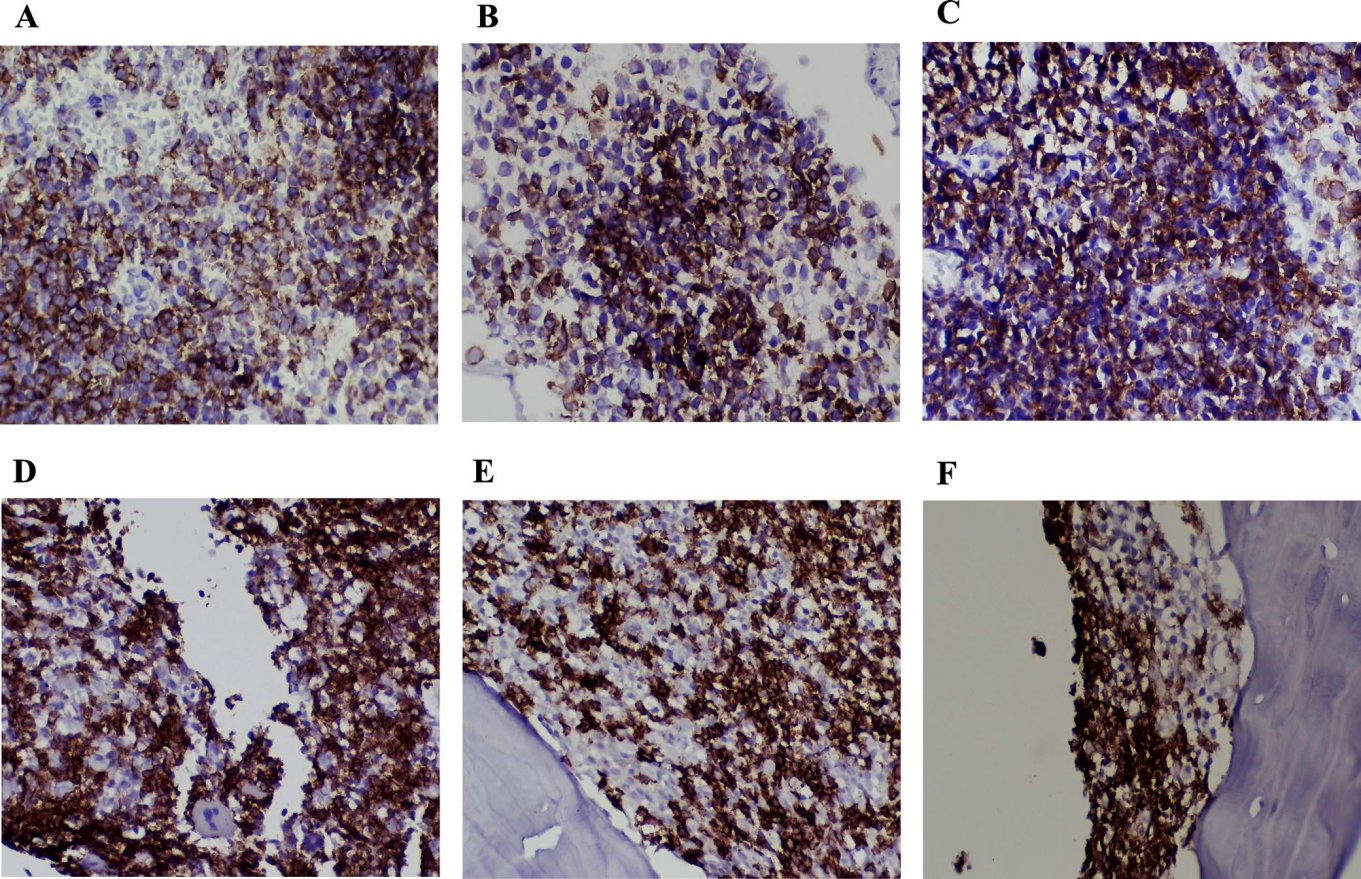
Immunohistochemistry (IHC) staining of breast and bone marrow. Breast; (A) CD5 positivity (400×), (B) CD20 positivity (400×), (C) CD23 positivity (400×). Bone Marrow; (D) CD5 positivity (400×), (E) CD20 positivity (400×), (F) CD23 positivity (400×).

Immunohistochemical staining on both the breast and bone marrow samples confirmed the presence of CLL cells. The neoplastic lymphocytes in the breast tissue were strongly positive for CD5, CD20, and CD23, supporting CLL infiltration (Figure 6A–C). Similarly, bone marrow IHC showed similar markers (Figure 6D–F).

## 4 Outcome and Follow‐Up

Following the diagnosis of leukemic infiltration in the breast, the patient received anti‐leukemic therapy. Given the patient's advanced age and the presence of extramedullary involvement, her prognosis remains poor. The disease was advanced, and the treatment approach focused on systemic control rather than local interventions. Regular clinical and radiological follow‐ups were recommended to monitor therapeutic response and disease progression.

## 5 Discussion

In this case report, we presented a 72‐year‐old woman with CLL who was diagnosed with breast involvement by CLL. Extranodal involvement of CLL is extremely rare, particularly in non‐lymphoid tissues like the breast. This involvement causes significant diagnostic and therapeutic challenges due to its rarity and potential to mimic primary breast malignancies.

Breast involvement by CLL can present differently in examination and imaging. In physical examination, it may appear as a mobile, palpable mass [10], or a hard, irregular mass with no skin or axillary involvement [11], or a lobulated and hard mass with bilateral axillary lymph node enlargement and diffuse thickened skin [12]. In mammography, it can represent a small nodular mass with regular margins and no microcalcifications [10], or a spherical opacity with mild irregular margins with a cluster of linear microcalcifications [11], or a hyperdense mass in the entire right breast [12]. MRI may show small lesions with irregular margins but no enhancement [13] and even occult mammography or sonography [8, 13]. Similarly, the lesion in our case appeared as a lobulated hyperdense mass in the right breast [12], and a smaller lobulated mass in the left breast with infiltrated lymph nodes on the left side; however, our case had no skin involvement.

Breast involvement by breast cancer has several differential diagnoses, including primary breast cancer and primary or secondary breast lymphoma, and patients with concurrent CLL and invasive ductal cancer have also been reported, which is called collision tumors [7, 14]. Patients with primary breast cancer may have symptoms such as nipple retraction, skin abnormalities, or shape alterations [15], and manifest as an irregular lesion with microcalcification or as a spiculated mass [16]. They arise from the breast ducts or lobules. Ductal carcinomas start in the ducts, whereas lobular carcinomas start in the lobules [17].

Lymphomas are the most common malignant hematologic tumors affecting the breast, and primary or secondary breast lymphoma occurs at a rate of 0.04%–0.7% [18]. An uncommon type of extranodal lymphoma, known as primary breast lymphoma, is characterized by the presence of a primary lesion inside the breast, either with or without the involvement of a regional node, but without the involvement of any other extra‐mammary locations. It includes a variety of histological subtypes, the most prevalent of which is diffuse large B‐cell lymphoma [19, 20]. It often manifests as a painless, quickly growing solitary tumor that cannot be clinically distinguished from breast carcinomas, either with or without ipsilateral axillary lymphadenopathy. Non‐specific radiographic findings of primary breast lymphoma coincide with those of breast cancer and often manifest as a painless, rapidly growing solitary tumor that cannot be clinically distinguished from breast carcinomas, either with or without ipsilateral axillary lymphadenopathy. Nonspecific radiographic findings of primary breast lymphoma coincide with those of breast cancer [21].

Approximately 17% of breast metastatic diseases are caused by secondary breast lymphoma, which is the most prevalent type of metastasis. The imaging phenotypes of primary and secondary breast lymphomas typically overlap with those of primary breast cancer, which complicates the prospective identification of breast lymphoma. Some examples of these nonspecific imaging findings include an iso‐ to hyperdense oval mass or mass on mammography, a hypoechoic or mixed‐echogenicity hypervascular mass on ultrasonography, an enhancing mass with type II kinetics on MRI, and a high fluorine 18‐fluorodeoxyglucose avidity on PET; these are some examples of these nonspecific imaging findings [22].

In this case, the patient presented with a palpable mass with a BI‐RADS classification of 4, suggestive of malignancy. Radiological features, including lobulated hyperdense masses in both breasts and axillary lymphadenopathy, have raised concerns about breast malignancy. Therefore, it is essential to perform fine‐needle aspiration cytology and histopathology to provide a conclusive diagnosis. Moreover, immunohistochemical tests, including cytogenetic marker testing, can help rule out breast metastases from hematological cancer [10]. This finding aligns with those of previous reports that emphasize the need for tissue diagnosis to distinguish CLL infiltration from primary breast cancer or other malignancies [10].

The management of CLL with breast involvement differs significantly from that of primary breast cancers. Breast mass treatment, such as endocrine therapy, immunotherapy, surgical excision, radiation therapy, and chemotherapy [23] is not the first‐line treatment for this type of involvement. Currently available treatments for CLL include chemotherapeutics, inhibitors of Bruton tyrosine kinase (BTK) (e.g., ibrutinib), an inhibitor of the anti‐apoptotic BCL2 protein (e.g., venetoclax), phosphoinositide‐3 kinase (PI3K) delta inhibitors, and anti‐CD20 monoclonal antibodies (e.g., rituximab) [24].

## 6 Conclusion

Given the rarity of this manifestation, more research and case studies are needed to better understand the clinical course, optimal management strategies, and long‐term outcomes in patients with CLL and breast involvement. Early recognition and accurate diagnosis are essential for improving patient outcomes and avoiding overtreatment in cases where systemic therapy is the appropriate approach.

## Author Contributions

**Ensiyeh Bahadoran:** investigation, visualization, writing – original draft, writing – review and editing. **Freidoon Solhjoo:** methodology, visualization, writing – review and editing. **Fatemeh SamieeRad:** conceptualization, investigation, supervision, visualization, writing – review and editing.

## Ethics Statement

The study was approved by the institutional review board and ethics committee. All applicable international, national, and/or institutional guidelines were followed and participant consent was obtained.

## Consent

Written informed consent was obtained from the patient to publish this report in accordance with the journal's patient consent policy.

## Conflicts of Interest

The authors declare no conflicts of interest.

## Data Availability

Data sharing is not applicable to this article as no new data were created or analyzed in this study.
